# Surgical and Patient Outcomes of Robotic Versus Conventional Laparoscopic Hysterectomy: A Systematic Review

**DOI:** 10.7759/cureus.16828

**Published:** 2021-08-02

**Authors:** Khadija Alshowaikh, Katarzyna Karpinska-Leydier, Jashvini Amirthalingam, Gokul Paidi, Anuruddhika I Iroshani Jayarathna, Divya Bala Anthony Manisha R Salibindla, Huseyin Ekin Ergin

**Affiliations:** 1 Obstetrics and Gynecology, California Institute of Behavioral Neurosciences & Psychology, Fairfield, USA; 2 Internal Medicine, California Institute of Behavioral Neurosciences & Psychology, Fairfield, USA; 3 General Medicine, California Institute of Behavioral Neurosciences & Psychology, Fairfield, USA; 4 Neurology, California Institute of Behavioral Neurosciences & Psychology, Fairfield, USA; 5 General Practice, California Institute of Behavioral Neurosciences & Psychology, Fairfield, USA

**Keywords:** robotic surgery, laparoscopic surgery, hysterectomy, operating time, estimated blood loss, length of hospitalization, survival analysis, cost

## Abstract

Hysterectomy is a commonly performed gynecologic surgery that can be associated with significant morbidity and mortality. However, the evolution of the surgical approach, from open to minimally invasive gynecologic surgery (MIGS), has substantially improved patient outcomes by reducing perioperative complications, pain, and length of hospitalization. The evident advantages and the approval of the da Vinci Surgical System by the Food and Drug Administration led to the exponential rise in the use of MIGS. In particular, robotic hysterectomy (RH) witnessed unparalleled popularity compared to other MIGS despite the lack of strong evidence demonstrating its superiority. Therefore, we conducted a systematic review of the literature to evaluate and compare various patient and surgical outcomes of RH with conventional laparoscopic hysterectomy (CLH), including operating time, estimated blood loss, length of hospitalization, overall complications, survival, and cost. Overall, the outcomes were comparable between RH and CLH except concerning cost. RH is significantly more expensive than CLH due to the higher costs of robotic equipment, including disposable instruments, equipment maintenance, and sterilization. Although RH demonstrated comparable outcomes and higher costs, its technical advantages such as improved ergonomics, three-dimensional view, a wider range of wristed mobility, mechanical lifting of robot’s hand, and greater stability might benefit patient subsets (e.g., obesity, large uterine weights >750 g). Therefore, large and multicentered randomized control trials are imperative to determine the most effective surgical approach between RH and other MIGS for different patient subsets.

## Introduction and background

Hysterectomy is one of the most common surgical interventions in gynecology for various benign and malignant indications including, leiomyoma, adenomyosis, abnormal uterine bleeding, endometriosis, uterine prolapse, and gynecologic malignancies [1]. A critical factor influencing post-hysterectomy morbidity is the surgical approach [2]. Traditionally, hysterectomy is performed via a midline laparotomy and is associated with significant morbidities, such as intraoperative organ injury, infections, hemorrhage, and wound dehiscence [3].

Over the past two decades, minimally invasive gynecologic surgery (MIGS) has revolutionized the science of gynecologic procedures, aiming to reduce perioperative complications and improve patient and surgical outcomes [4]. MIGS includes conventional laparoscopic surgery and robotic surgery. Conventional laparoscopic surgery utilizes small incisions to manipulate tissues with endoscopic cameras and long instruments surgically [4]. In contrast, robotic surgery allows a computer interface between the surgeon and patient, employing more technologically advanced equipment with three-dimensional (3D) viewing, commonly controlled from a remote console [4].

The use of robotic surgery in gynecology has gained popularity since the approval of the da Vinci Surgical System by the Food and Drug Administration in 2005 [5]. The first simple hysterectomy using robotic technology was performed nearly two decades ago [6]. Since then, an estimated three million gynecologic robotic surgeries have been performed worldwide. The use of robotic hysterectomy (RH) increased by 1000%, from 0.5% to 9.5%, between 2007 and 2010 [5,7]. Moreover, the rise in conventional laparoscopic hysterectomy (CLH), from 24.3% to 30.5%, was slower than RH in the same period [7]. 

Today, MIGS has become the standard of care given its clear benefits compared to the open surgical approach [8]. MIGS results in decreased perioperative complications, blood loss, post-operative pain, faster recovery, and shorter hospitalization when compared to laparotomy [8,9]. Moreover, the overall quality of life, patient satisfaction, and post-operative social functioning appear to be significantly superior after minimally invasive hysterectomy [10].

Despite level-one evidence showing advantages of minimally invasive hysterectomy over laparotomy, scant data compare outcomes between different MIGS [5,11,12]. Currently, gynecologists choose the surgical approach based on their personal preference [2]. Thus, we aim to compare patient and surgical outcomes of RH versus CLH to establish whether a particular MIGS approach is superior for hysterectomy. We also investigate if the rapid increase in popularity of RH is matched with better therapeutic outcomes than CLH for benign and malignant gynecologic conditions.

## Review

Methods

Search Strategy

A standard methodology was conducted following the Preferred Reporting Items for Systematic Reviews and Meta-Analyses (PRISMA) guidelines [13]. A systematic review of the literature was performed by electronic search of the databases PubMed/Medline, Embase, and Scopus from inception to April 29, 2021. The keywords “robotic surgery,” “laparoscopic surgery,” “hysterectomy,” and the Boolean term “and” were used to find relevant studies. Medical Subject Heading (MeSH) terms and keywords were also used in combination to populate thematic sets. Automated filters on language, time, gender, and article type were applied, and duplicate articles were eliminated. Inclusion criteria comprised: (1) articles comparing any surgical or patient outcomes of RH and CLH, (2) adult female population undergoing hysterectomy for benign or malignant conditions, (3) articles in the English language, (4) publication dates between 2016 and 2021, (5) study design is a classical article, clinical study, journal article, observational study, or comparative study, and (6) the study was published as a peer-reviewed manuscript. Gray literature, books, documents, case series, case reports were excluded. Two independent researchers (K.A. and K.K.) manually reviewed all titles, abstracts, and full texts to determine eligibility, with disagreements resolved by mutual discussion and consensus. Table 1 and Table 2 display the search strategy results using MeSH terms and keywords. 

**Table 1:** Search strategy with MeSH terms. MeSH: medical subject headings; Majr: major topics. Note: The data shown in the table contains duplicates and articles that did not meet eligibility criteria, which were later removed.

MeSH terms	Total number of articles	Number of articles after the application of automated search filters
Laparoscopy OR Laparoscopic assisted OR ("Laparoscopy/adverse effects"[Majr] OR "Laparoscopy/complications"[Majr] OR "Laparoscopy/economics"[Majr] OR "Laparoscopy/mortality"[Majr] OR "Laparoscopy/therapeutic use"[Majr] AND Robotic surgery OR Robotic OR robotic assisted OR ( "Robotic Surgical Procedures/adverse effects"[Majr] OR "Robotic Surgical Procedures/economics"[Majr] OR OR "Robotic Surgical Procedures/mortality"[Majr] OR "Robotic Surgical Procedures/therapeutic use"[Majr] AND Hysterectomy OR ("Hysterectomy/adverse effects"[Majr] OR "Hysterectomy/complications"[Majr] OR "Hysterectomy/mortality"[Majr] OR "Hysterectomy/therapeutic use"[Majr] OR "Hysterectomy/therapy"[Majr])	4,545	983
((Robotic[MeSH Major Topic]) AND (laparoscopic[MeSH Major Topic])) AND (hysterectomy[MeSH Major Topic])	89	89
(("Robotic Surgical Procedures/adverse effects"[MeSH]) AND "Laparoscopy/adverse effects"[MeSH]) AND ("Hysterectomy/therapeutic use"[MeSH] OR "Hysterectomy/therapy"[MeSH])	39	33
(("Robotic Surgical Procedures/economics"[MeSH]) AND "Laparoscopy/economics"[MeSH]) AND ( "Hysterectomy/therapeutic use"[MeSH] OR "Hysterectomy/therapy"[MeSH])	4	4
((Robotic[MeSH Terms]) AND (laparoscopy[MeSH Terms])) AND (gynecology[MeSH Terms])	32	12
((("Laparoscopy/therapeutic use"[MeSH] OR "Laparoscopy/therapy"[MeSH])) AND ("Robotic Surgical Procedures/therapeutic use"[MeSH] OR "Robotic Surgical Procedures/therapy"[MeSH])) AND ("Hysterectomy/adverse effects"[MeSH] OR "Hysterectomy/complications"[MeSH])	39	33
(((("Laparoscopy/therapeutic use"[MeSH] OR "Laparoscopy/therapy"[MeSH])) AND ( "Robotic Surgical Procedures/therapeutic use"[MeSH] OR "Robotic Surgical Procedures/therapy"[MeSH]))) AND "Hysterectomy/mortality"[MeSH]	1	1

**Table 2:** Search strategy with keywords. Note: The data shown in the table contains duplicates and articles that did not meet eligibility criteria, which were later removed.

Keywords	Database	Total number of articles	Number of articles after the application of automated search filters
Robotic surgery and laparoscopic surgery and hysterectomy	PubMed/Medline	733	295
	Scopus	1,038	387
	Embase	2,436	397

Risk of Bias Assessment

The 30 studies that met the inclusion criteria underwent rigorous quality appraisal. The Newcastle-Ottawa scale was used to assess observational/non-randomized controlled trials. The final analysis included observational studies of high quality, scoring greater than seven. Randomized Control Trials (RCTs) were assessed using the Cochrane Bias assessment tool. Only low-risk bias trials are included in this review.

Results

Search Outcome 

**Figure 1:**
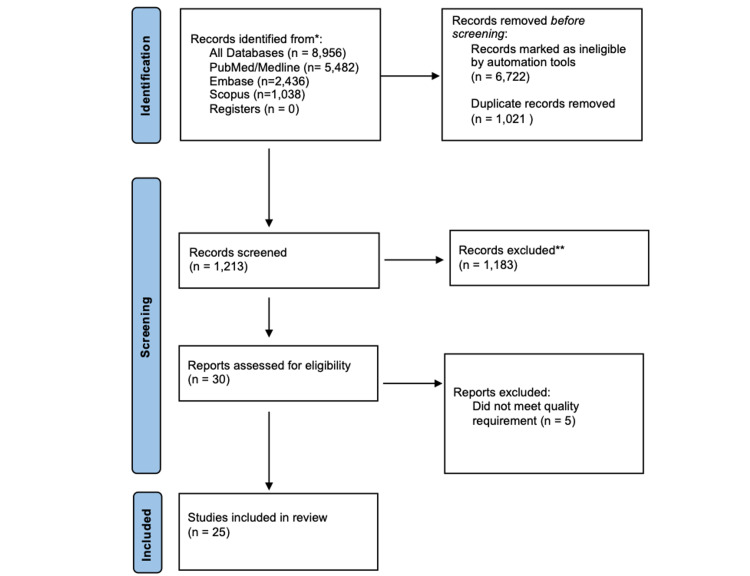
PRISMA flowchart diagram. PRISMA: Preferred Reporting Items for Systematic Reviews and Meta-Analyses. *: Electronic databases were used to search for records. **: Records were excluded if publications did not compare surgical or patient outcomes between conventional laparoscopic hysterectomy and robotic hysterectomy.

In total, 8,956 records were identified via the initial search of the afore-mentioned databases: 4,207 articles identified with keywords in combination and 4,749 articles using the MeSH strategy. The application of automated search filters yielded 2,234 studies with 1,021 duplicate articles that were then removed using EndNote Basic (Clarivate, Boston, USA). The remaining 1,213 studies were screened for relevance, following which 1,183 articles were removed. Lastly, five articles were further excluded after quality assessment, resulting in 25 articles in this review. Figure 1 details the PRISMA flowchart diagram of literature retrieval for this systematic review. 

Study Characteristics

The 25 finalized articles consisted of two RCTs, one case-control study, and 22 cohort studies. All included articles were published in peer-reviewed journals on or after 2016 and compared the patient and surgical outcomes between RH and CLH for benign and/or malignant gynecologic indications. All included studies were conducted in tertiary academic hospitals across 13 countries. The overall sample size for this systematic review is 57,697 participants, 16,826 of which underwent robotic or robotic-assisted hysterectomy while 40,871 participants had a laparoscopic hysterectomy.

The main outcomes of reviewed studies are summarized in Table 3. 

**Table 3:** Study findings of included articles comparing RH and CLH. RH: robotic hysterectomy; CLH: conventional laparoscopic hysterectomy.

Author	Year	Type of study	Study purpose	Outcome
Aiko et al. [14]	2020	Retrospective cohort	Compare the short-term outcomes of CLH and robotic assisted surgery for early stage endometrial cancer.	The operative time and blood loss was significantly higher for the robotic group without a significant difference in the number of lymph nodes.
Barrie et al. [15]	2016	Retrospective cohort	Compare intraoperative and postoperative surgical complications between RH and CLH for the management of endometrial cancer.	There was no difference in the rate of major complication between RH and CLH using the Clavien–Dindo system. However, RH had significantly lower rate of minor complications and conversions compared to CLH.
Beck et al. [16]	2018	Retrospective cohort	Compare patient outcomes by surgical approach for endometrial cancer.	RH is a safe alternative to CLH surgery for the treatment of endometrial cancer. RH resulted in fewer early readmissions.
Borahay et al. [17]	2018	Retrospective cohort	Compare the outcomes of total abdominal hysterectomy, RH and CLH in obese patients with benign conditions.	Minimally invasive surgery is safe in obese patients with less blood loss, fewer intraoperative complications and shorter hospital compared to total abdominal hysterectomy. No significant differences were noted for later postoperative complications (>6 weeks).
Brunes et al. [18]	2021	Prospective cohort	Study the effects of obesity on perioperative and postoperative outcomes of patients undergoing RH versus CLH.	The use of robotic surgery in obese patients may lower conversion rates to laparotomy and intraoperative bleeding.
Chen et al. [19]	2019	Retrospective cohort	Analyze the perioperative conditions, complications, short-term and long-term effects of radical RH and CLH.	Radical RH is associated with significantly less operative time and blood loss compared to CLH. The complication rates, overall survival and progression-free survival were similar.
Deimling et al. [20]	2016	Randomized control trial	Compare the operative time between CLH and RH.	The operative time was comparable when performed by an experienced surgeon.
Eoh et al. [21]	2021	Retrospective cohort	Compare RH and CLH for managing endometrial carcinoma.	RH is safe and comparable to other surgical approaches for the management of low-risk endometrial cancer patients.
Fanfani et al. [22]	2016	Case-control	Compare the feasibility and safety of RH and CLH in benign and early malignant gynecologic disease.	RH is a safe, feasible and valid option for hysterectomy in patients with benign and early malignant gynecologic disease.
Gracia et al. [23]	2020	Retrospective cohort	Compare perioperative outcomes and complications in robotically assisted laparoscopy and standard laparoscopy in the treatment of endometrial cancer by body mass index (BMI).	Robotic assisted surgery is superior in treating obese women with endometrial cancer by reducing blood loss and conversion rates.
Gueli Alleti et al. [24]	2016	Retrospective cohort	Compare the surgical and clinical outcomes of RH and CLH in patients with early-stage endometrial cancer.	Based on operative outcomes and complication rates, RH is feasible and safe for early-stage endometrial cancer.
Gungor et al. [25]	2017	Retrospective cohort	Compare the perioperative parameters of single port RH and single port CLH.	Single port RH and single port CLH are comparable and safe in terms of operative time, conversion to laparotomy or multiport surgery, complication rates and postoperative results.
Han et al. [26]	2019	Retrospective cohort	Compare the safe and efficacy of RH and CLH for the treatment of cervical cancer using multivariate regressions.	RH has a shorter hospital stay. No difference was noted relating to blood loss or postoperative complications. RH is a safe and feasible alternative procedure.
Jørgensen et al. [27]	2019	Prospective cohort	Evaluate the survival of women with early-stage endometrial cancer undergoing robotic minimally invasive surgery.	No significant survival difference between robotic and laparoscopic minimally invasive surgery.
Johnson et al. [28]	2017	Retrospective cohort	Compare outcomes of robotic, laparoscopic, and open procedures for endometrial cancer.	Laparoscopic cases were shorter than robotic and open cases with fewer conversions. This could be due to reduction in node dissection performed.
Mäenpää et al. [29]	2016	Randomized control trial	Compare RH and CLH for the management of endometrial cancer.	RH had lower operative time compared to CLH. Other surgical outcomes were comparable between the two approaches.
Moawad et al. [30]	2017	Retrospective cohort	Compare the cost and outcomes of RH and CLH across different uterine weights.	RH for uteri weighing >750g may be associated with shorter operative time and improved cost profile compared to CLH.
Netter et al. [31]	2020	Prospective cohort	Compare the procedure characteristics of CLH and RH for gynecologic cancers in the context of enhanced recovery program (ERP).	Postoperative complications were similar between the groups.
Ngan et al. [32]	2017	Retrospective cohort	Compare patient perioperative complications and cost of CLH with RH for uterine leiomyomas.	Perioperative outcomes are comparable between the approaches with greater direct costs associated with RH.
Nie et al. [33]	2017	Retrospective cohort	Compare surgical outcomes of radical RH with CLH for early stage cervical cancer.	RH is superior to CLH with regards to operating time, blood loss, length of hospitalization (LOH), duration of bowel function recovery, and postoperative complications. RH is a safe and feasible alternative outcome.
Oyama et al. [34]	2018	Retrospective cohort	Compare short-term outcomes of radical CLH and RH for early-stage cervical cancer.	The operative time, blood loss and number of removed lymph nodes was higher for the robotic group. However, the differences between the groups seem to be within clinically acceptable range.
Pellegrino et al. [35]	2017	Prospective cohort	Compare the clinical and oncological outcomes of radical RH and CLH in patients with cervical carcinoma.	RH is associated with decreased blood loss. The operative time, hospital stay, and complication stay were similar between the two groups.
Rajadurai et al. [36]	2018	Retrospective cohort	Compare the outcomes of patients undergoing RH and CLH.	Equivalent morbidity, post-operative pain, opioid use and hospital stay were noted.
Sinha et al. [37]	2019	Retrospective cohort	Compare the outcomes of RH and CLH for larger uteri >16 weeks.	RH is safe with lower blood loss even with high body mass index, extensive adhesiolysis and difficult bladder dissection.
Takmaz and Güngör [38]	2020	Retrospective cohort	Compare early surgical outcomes of RH versus CLH for benign disease.	Early surgical outcomes between the two approaches were comparable in terms of blood loss, first gas discharge and hospital stay. The operative time was longer for RH.

Discussion

Hysterectomy is a common gynecologic surgery performed for benign and malignant indications [1,37]. The evolution of the surgical approach in gynecology to MIGS drastically reduced the perioperative morbidity associated with hysterectomy [31]. More recently, the introduction and validation of robotic surgery have increased the widespread use of MIGS and allowed for better patient and surgical outcomes [27]. Additionally, the implementation of enhanced recovery programs (ERPs) allowed healthcare providers to evaluate and further improve surgical quality and patient health [31]. ERPs aim to decrease hospitalization length without increasing perioperative complications and readmission rates, creating an optimal and standardized patient recovery environment [31]. Despite the strong evidence suggesting enhanced surgical and patient outcomes of MIGS compared to open approaches, the benefits of RH over CLH are still debated [31]. Therefore, our systematic review compares various outcomes between RH and CLH.

Operating Time (OT)

The OT is influenced by multiple variables, including patient-related factors, surgeon expertise, surgical technique, and approach [30]. Obesity, higher age, increased uterine weight, and extensive adhesions are patient-related factors that can increase surgical complexity and, subsequently, the OT [30,31]. Brunes et al. found that the frequency of hysterectomies lasting more than two hours was at least four-fold higher with CLH than RH in obese patients [18].

Moreover, the longer OT in CLH noted in some included studies can be explained by the higher numbers of obstetrics and gynecology residents involved in CLH training than RH [15]. Extensive experience in CLH subsequentially reduces RH OT, supported by a shorter learning curve [24,36]. It is, therefore, evident that the surgeon’s expertise is an essential factor in determining the OT [15].

Additionally, instrumental preparation, such as docking time in RH, which is the fixation of robotic arms to the ports, can increase the OT [38]. However, the docking time becomes progressively shorter as the surgical team gains experience [29]. The type of hysterectomy, supplementary procedures at the time of hysterectomy, morcellation, and the presence of a large and dedicated surgical team also influence the OT [18,30].

Of the studies reviewed, nine showed longer OT in RH, eight showed longer OT in CLH, and four showed no significant differences. The mean overall OT for RH ranged from 75.42 to 306.03 minutes, while the CLH OT ranged from 53.18 to 323.25 minutes. Due to the various elements affecting OT and the difficulty in controlling confounding variables, the OT of CLH was comparable to RH in this systematic review.

Estimated Blood Loss (EBL)

Seventeen studies included in this review measured the EBL in CLH and RH; most studies showed no statistically significant differences between the two groups. The EBL ranged from 50 to 237 ml and 50 to 230.5 ml in the RH and CLH groups, respectively. Aiko et al. and Oyama et al. suggest that the use of different instrumentation in the two approaches can affect the EBL [14,34]. The Probe Plus II (Ethicon Endo-Surgery, Inc., Blue Ash, OH, USA) is a suction irrigator probe with a built-in monopolar electrode used in CLH [14,34]. This device can restore quick homeostasis by immediately detecting bleeding points without exchanging forceps [14,34]. There is no equivalent instrument used in RH [14,34]. Moreover, significant differences in the EBL between RH and CLH could possibly be detected in patients with a high body mass index (BMI) [23]. Gracia et al. found that the EBL reduction is more significant in obese patients undergoing RH than in normal weight or overweight patients compared to CLH [23]. 

Length of Hospitalization (LOH)

LOH is listed by the Agency for Health-care Research and Quality as a vital patient safety indicator [16]. Longer LOH raises morbidity by increasing the risk of nosocomial and surgical site infections, readmission rates, and reducing the short-term quality of life [16]. Our review suggests no clinical or statistical difference in the LOH between RH and CLH. The median LOH ranged between 1 to 18.57 days in the RH group and 1 to 18.23 days in the CLH group. Studies demonstrating significant LOH differences between the two approaches attributed these differences to factors other than surgical approach, including reimbursement issues, availability of insurance, number and width of incisions, age, or BMI [14,23,31]. 

Overall Complication Rate

Fifteen reviewed studies measured the complication rates in RH and CLH: of these, 11 studies found no statistical difference between the approaches and one study showed a higher complication rate in CLH; additionally, three studies found differences after complication classification. Barrie et al. and Chen et al. both found no significant differences in intraoperative complications but showed substantial reductions in postoperative complications for the RH patients [15,19]. Early postoperative complications were mainly wound and urinary tract infections, while long-term postoperative complications included lymphatic drainage disorders [19]. Meanwhile, Ngan et al. suggest that some postoperative complications, such as respiratory failure, are higher in RH patients due to higher risks of facial and upper-airway edema resulting from longer OT in the steep Trendelenburg position [32]. 

Furthermore, complication rates increase with more extensive surgery, involvement of learners, patient’s age, and obesity [15]. Brunes et al. show that the overall complication frequency within one year of hysterectomy, particularly wound infections, was higher in women with obesity class II-III [18]. These confounding factors influence the overall complication rate regardless of the surgical approach used and have to be controlled for to accurately compare the complication rates between RH and CLH [15,18].

Survival

The index surgery, age group, and modified Charlson comorbidity index are significant predictors of survival [21]. All but one study in this review showed no statistically significant differences in patient survival between RH and CLH even after adjustment by the Kaplan-Meier survival curve and the Cox proportional hazards [27]. Pellegrino et al. attributed the higher overall survival rate in the RH group to the low volume of patients in the CLH group resulting in calculation bias [35]. 

Cost

The widespread use of RH has been limited by expense [34]: increased costs are attributable to the price of robotic instruments, instrument sterilization and maintenance, use of disposable instruments, patient and robot draping, and the OT [30]. RH remains 1.43 times more costly, with a median difference of $12,893, even after adjustment for age, LOH, and conversion to laparotomy [32]. However, RH proved to be more profitable in complex procedures, obese patients, and in uterine weights >750 g [30,31]. The price per patient is also expected to drop with increased RH use and surgeon experience [33]. 

Advantages of RH Compared to CLH

RH provides a 3D view, greater range of movement with wristed instruments, improved dexterity, higher stability, and fatigue-resistant properties allowing for better visualization and a more precise surgical technique [17,26,29]. Whereas, CLH uses the abdominal wall as leverage for movement, limiting mobility and causing more tissue damage at the abdominal wall [37]. Additionally, the mechanical lift of the robot’s arms enables better maintenance of the field of view [14]. The learning curve for RH, defined by the setup time, console time, and the number of cases required for a surgeon’s OT to stabilize, is approximately 50 cases [36]. The learning curve is shorter in RH than CLH, which means that less extensive practice is needed to master the procedure [19]. 

Disadvantages of RH Compared to CLH

The robotic system is both complex and large. It comprises three components: a surgeon console, a patient card, and an endoscopic tower, therefore, requiring a large operating room and trained healthcare workers to operate the system [38]. Moreover, the port incisions in RH are more numerous and larger, leading to cosmetically unfavorable results and increases multi-port-related complication risk such as hematoma, herniation, vascular or visceral injury, wound infection, and pain [25,38]. Single-port RH has been investigated by a limited number of studies but shows more promising surgical outcomes compared to single-port CLH [24]. Additionally, the higher cost of RH discussed earlier and the absence of tactile feedback is another major limitation of robotic surgery [20].

Strengths and limitations

The strength of this study lies in its large collective sample size of 57,697 participants who underwent either CLH or RH. A large sample size is essential to assess the representativeness and generalization of the study sample to the whole population. Another strength is the bias risk assessment conducted to appraise the included articles; only high-quality studies were included. Limitations of this systematic review include the paucity of large RCTs and case-controls reviewed due to a deficiency in current literature. Since most reviewed studies were retrospective cohorts, selection bias could not be eliminated. Moreover, surgeon bias and clinical heterogeneity could not be excluded in this review due to the inclusion of patients with varied gynecologic diagnoses and studies from numerous centers in different countries. 

## Conclusions

In conclusion, our review suggests no difference in surgical and patient outcomes between RH and CLH relating to OT, EBL, LOH, overall complications, and survival. However, the RH cost remains to be significantly higher compared to CLH. RH is a safe and comparable alternative for CLH, possibly providing greater benefits in patients with obesity and large uterine weights. It is essential to interpret these analyses with precaution as RH is a relatively new technology in its evolutionary phase. Large multicentered RCTs are required to eliminate bias and provide sufficient evidence to establish the superiority of a MIGS approach in hysterectomy.
